# Essential epidemiological mechanisms underpinning the transmission dynamics of seasonal influenza

**DOI:** 10.1098/rsif.2011.0309

**Published:** 2011-06-29

**Authors:** James Truscott, Christophe Fraser, Simon Cauchemez, Aronrag Meeyai, Wes Hinsley, Christl A. Donnelly, Azra Ghani, Neil Ferguson

**Affiliations:** 1 MRC Centre for Outbreak Analysis and modelling, Department of Infectious Disease Epidemiology, Imperial College, London W2 1PG, UK

**Keywords:** seasonal influenza, multiple strains, model comparison

## Abstract

Seasonal influenza has considerable impact around the world, both economically and in mortality among risk groups, but there is considerable uncertainty as to the essential mechanisms and their parametrization. In this paper, we identify a number of characteristic features of influenza incidence time series in temperate regions, including ranges of annual attack rates and outbreak durations. By constraining the output of simple models to match these characteristic features, we investigate the role played by population heterogeneity, multiple strains, cross-immunity and the rate of strain evolution in the generation of incidence time series. Results indicate that an age-structured model with non-random mixing and co-circulating strains are both required to match observed time-series data. Our work gives estimates of the seasonal peak basic reproduction number, *R*_0_, in the range 1.6–3. Estimates of *R*_0_ are strongly correlated with the timescale for waning of immunity to current circulating seasonal influenza strain, which we estimate is between 3 and 8 years. Seasonal variation in transmissibility is largely confined to 15–30% of its mean value. While population heterogeneity and cross-immunity are required mechanisms, the degree of heterogeneity and cross-immunity is not tightly constrained. We discuss our findings in the context of other work fitting to seasonal influenza data.

## 1. Introduction

Seasonal influenza causes significant levels of morbidity and mortality around the world each year, yet its dynamics and the annual sequence of pathogen subtypes are hard to predict [1]. Understanding the mechanisms underlying the annual behaviour of influenza and their sensitivity to parameters is important for the forecasting and control of seasonal epidemics and also in assessing the effect of the introduction of novel antigenic strains. The 2009 H1N1 pandemic is a recent example [2]. The initial outbreak of this novel H1N1 strain occurred in spring and summer, ‘out of season’ in the Northern Hemisphere. Uncertainty with regard to intensity of transmission during summer substantially complicated forecasting of the likely trajectory of the epidemic over the following months.

Many respiratory transmissible diseases exhibit seasonal epidemics. Annual periodic forcing causes a wide range of oscillatory epidemic behaviour, as illustrated by incidence rates for measles and pertussis [3–5]. The source of seasonal forcing in those cases is largely attributed to annual variations in the intensity of contact between children, but a range of other mechanisms have been proposed. In the case of influenza, two recent hypotheses have centred on variation in vitamin D levels and air humidity [6,7].

Influenza dynamics are additionally complicated by considerable antigenic diversity in human influenza viruses. Within each of the two influenza A subtypes (H3N2 and H1N1) co-circulating prior to 2009, continual evolution selecting for antigenic novelty was seen [8]. The dynamics of intra-subtype evolution is characterized by relatively stable strains periodically replaced by antigenically distinct types in punctuated evolution events [8–10]. In addition, there is evidence of immune-mediated competition between subtypes [10]. These mechanisms combine to generate complex seasonal behaviour, featuring a range of annual attack rates (AARs) and epidemic durations and alternating sequences of dominant annual subtypes and strains [11]. Detailed and computationally intensive simulations are required to fully integrate the epidemiological and genetic aspects of long-term behaviour [10,12]. However, strain-specific data with sufficient temporal resolution to fit such a model are not available. We therefore adopt a simplified description here and model two weakly interacting subtypes, with evolution within each subtype being represented by a gradual loss of immunity of the previously exposed host population, a so-called SIRS (susceptible–infected–recovered–susceptible) model. While use of SIRS models to represent intra-subtype evolution is not uncommon [13–15], previous models of influenza time series have not accounted for dynamics generated by multiple interacting subtypes.

In this paper, we identify a set of key features characterizing seasonal influenza-like illness (ILI) incidence time series and use these to define information measures for the distance between a transmission model and empirical observations. Use of summary statistics (rather than attempting to directly fit models to time-series data) adds robustness to variations in reporting (much ILI is not even caused by influenza) and short-term fluctuation in the incidence data. Our aim is not to precisely match the incidence time series, but to find broad regions of parameter space which are consistent with the characteristics of seasonal flu epidemics and hence identify which epidemiological mechanisms are essential and acceptable ranges for their parameters. For this purpose, an elaborate model and detailed data are not necessary.

**Figure 1.**
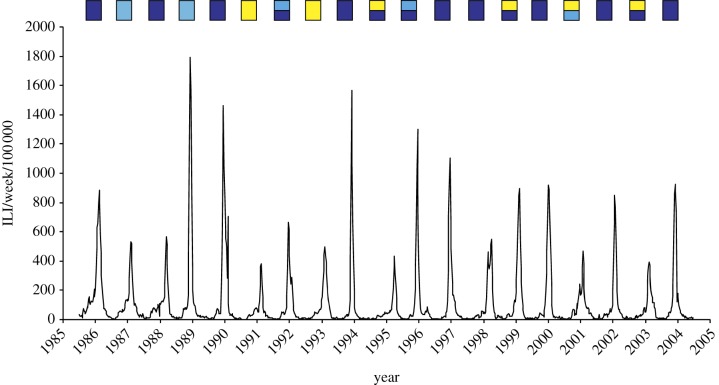
Medically attended influenza-like illness weekly incidence in France, 1984–2004. The dominant types and subtypes of circulating influenza viruses circulating in France are shown in the rectangles (after Denoeud *et al*. [11]). Dark blue, A/H3N2; light blue, A/H1N1; yellow, B.

We use the following features of the time series to characterize seasonal influenza incidence in temperate countries of the Northern Hemisphere:

— *Epidemic duration*. The vast majority of seasonal influenza outbreaks are observed between the extremes of mid-November and the end of April [11]. Since a background rate of ILI incidence is present throughout the year, duration is difficult to define precisely. The range of values quoted for the duration of an annual epidemic (described with the acronym ADE in this paper) is typically between three and 16 weeks [1,16].
— *Attack rate*. The proportion of the population infected in a year, which we term the AAR, is very difficult to determine, as a significant proportion of infections are asymptomatic and only a proportion of symptomatic cases seek healthcare. Hence sentinel data on ILI can only give relative information, such as the fractional variation in incidence over time. In addition, measures such as ILI are non-specific for influenza and can be caused by a variety of respiratory pathogens. That said, ILI data from France and the UK indicate a standard deviation for AAR across successive years of approximately 40 per cent of the mean attack rate [17,18]. Results from serological and virus isolation data from closely monitored populations indicate an AAR range of approximately 10–20% for seasonal influenza, rising to 30 per cent or more for influenza pandemics [1,17]. Information on consultation rates for known cases also suggests a mean AAR of around 15 per cent [13,18,19].
— *Periodic behaviour*. Seasonal influenza is characterized by annual outbreaks in temperate countries in the sense that there is almost invariably a marked seasonal increase in case rate during the winter months. However, it cannot be said to be periodic in the sense that successive outbreaks are comparable in magnitude and form with each other. In this sense, seasonal influenza has no clear periodicity (annual, biennial and triennial) and contrasts with the behaviour of diseases such as measles, which has a pronounced biennial structure in the pre-immunization period [5]. The metrics that we use to compare model and time-series behaviour are able to pick up this characteristic aperiodicity.
— *Strain variation*. Virus isolation studies show that individual seasonal epidemics are usually dominated by a single type and/or subtype, although others may be present at low levels [13]. Successive seasons are usually dominated by different types and subtypes, although often the same strain is present for several seasons (figure 1).

Seasonal influenza is characterized by annual outbreaks in temperate countries in the sense that there is almost invariably a marked seasonal increase in case rate during the winter months. However, it cannot be said to be strictly periodic in the sense that successive outbreaks are comparable with each other in magnitude and duration. In this sense, seasonal influenza has no clear periodicity and contrasts with the behaviour of diseases such as measles, which has a pronounced biennial structure in pre-immunization period [5].

## 2. Model and fitting technique

**Figure 2.**
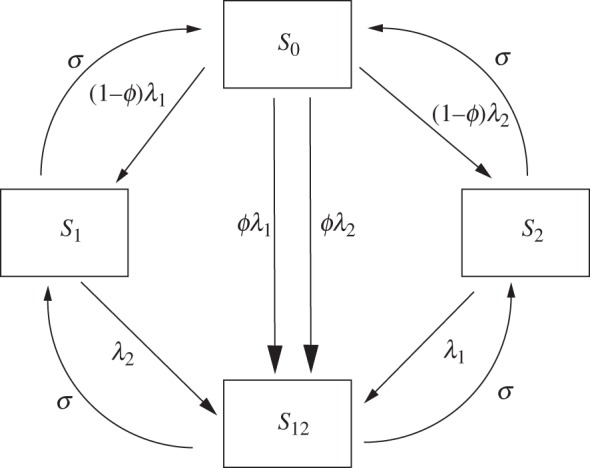
Immune state transitions, with rates per individual, between classes in the two strain model.

We use a deterministic SIRS compartmental model, to which we have added a number of refinements to capture critical aspects of influenza infection and immunity. The basic dynamics of infection and immunity in the model are
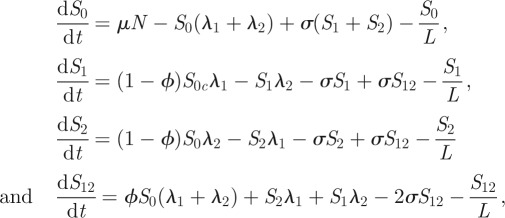
where *λ*_*j*_ is the force of infection of strain *j* and *S*_*n*_ is the part of the population that is immune to strain *n*. Infected individuals recovering from strain *n* enter a class entirely immune to *n* (e.g. *S*_0_ → *S*_1_, *S*_1_ → *S*_12_). Immunity to a given strain is lost at a rate *σ*. Figure 2 illustrates the flows of individuals between the various immunity classes. The model is further complicated by stratification by age (children and adults) with heterogeneous mixing, differential infectiousness and susceptibility by age and a realistic infectiousness profile. The full details of the model are described in the electronic supplementary material.

The effect of seasonal forcing is included through a time-varying contact parameter,
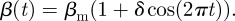


We express this variation in terms of peak *R*_0_ and relative amplitude of variation throughout. The sinusoidal form is a good match for variation in infectiousness as a function of absolute humidity in temperate regions [14], but we also examine the effect of using school terms as a seasonal driver using step-function forcing (see the electronic supplementary material). In determining feasible ranges for these parameters, we reviewed the range of estimates that exist for *R*_0_. The majority of these are calculated for the major global epidemics (1918, 1957, 1968), since the antigenic novelty of pandemic viruses means an assumption of a serologically naive population can be made, making analysis simpler. For seasonal influenza, knowledge of population susceptibility is necessary to estimate *R*_0_ (as opposed to the effective reproduction number, *R*). Estimated values for the 1918 pandemic range from 1.3 to 2.8 [20]. Similar values are found for the 1957 and 1968 epidemics [21]. These values also correspond well with those from studies of seasonal influenza, giving winter *R*_0_ of 1.7 and school holiday *R*_0_ of 1.4 [16].

The periodic forcing of systems of ordinary differential equations leads to a rich variety of behaviour, characterized by solutions with periods that are multiples of the forcing period (see [22] for SEIR example). Typically, smooth variation of the parameters can lead to sudden changes in the periodicity of the stable behaviour of the system. As will be seen in §3, the goodness of fit of the model is strongly dependent on the periodicity of the model's solution.

We represent the generation time for the pathogen by an Erlang distribution with shape parameter *k* = 4 and mean 1/*α*, where *α* = 2.7 days [20,23]. Our model incorporates two strains of influenza, for instance, representing H1N1 and H3N2, to try and capture aspects of the co-circulation of multiple influenza types and subtypes [11]. There are four immune states for individuals in the model; entirely susceptible, immune to either strain 1 or 2 and immune to both strains. The formulation allows for the inclusion of a basic cross-immunity mechanism, whereby an individual infected with either strain has a probability, *ϕ*, of becoming immune to the other as well (assuming that this was not already the case) The flow between different immune states is illustrated in the electronic supplementary material. We note that this cross-immunity response is different from the short-term non-specific response with regard to influenza strains considered elsewhere [10], though cross-immunity is assumed to wane at the same rate as strain-specific immunity.

Our model also includes a mechanism for loss of immunity, returning individuals to a susceptible state. Surveillance data show that human influenza strains can be grouped into clusters within each of which there is a high level of cross-immunity, but between which cross-immunity is much lower [8]. The appearance of a new cluster therefore corresponds to a step change in the susceptibility of the population to the current strain. Our model caricatures this process with a timescale, *D*, for resistant individuals to become susceptible to the current strain again. As antigenically distinct clusters appear every 2–8 years [8,24], this is our expected range for values of *D*.

There is evidence from contact studies and from modelling of influenza epidemics that infectious contact between individuals is highly assortative and age-dependent, with the highest rates among school-age children [16,25,26]. We include these effects by stratifying the population into children (less than or equal to 14 years) and adults (more than 14 years) and employing a mixing matrix to describe contact between the two groups. The degree of assortativity is controlled by the parameter, *θ*, and can be varied between random mixing (*θ* = 0), where groups contact each other proportional to the fraction of the population they represent, and wholly assortative (*θ* = 1) where each group mixes only with itself. Differences in intensity of contact are captured by relative susceptibility and infectiousness parameters, *ρ* and *ψ* (see the electronic supplementary material for details).

To assess the quality of fit of the model behaviour to the data, we compare the distribution of key features in the time-series data with those generated by the epidemic model using the Kullback–Leibler (KL) information distance. We use normal distributions to characterize the empirical distributions of AAR and epidemic duration across a number of years. As discussed above, ignorance of the reporting rate makes it hard to know the underlying ‘real’ infection rate and also makes it difficult to compare reported incidence collected under different surveillance systems. In order to compare the data from the UK and France, we assume constant reporting rates for the UK and French surveillance systems, respectively, and scale the reported values linearly such that each has a mean AAR of 15 per cent (see the electronic supplementary material). Both dataset yield standard deviations of around 35 per cent of mean value for AAR and 11 ± 2 weeks for epidemic duration.

We calculate the KL information distance between model and data, *I*, for each of the key features as follows:
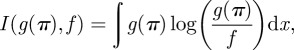
where *f* is the distribution taken from the data, *g* is the approximate distribution of the same feature recovered from the model over many simulated years and *π* is a vector of model parameters. (See the electronic supplementary material for implementation.) The overall measure of goodness of fit used is the unweighted sum of the information distances for AAR and duration.

We explore parameter space to identify regions where model behaviour most closely resembles empirical patterns. Although a simplified description of the epidemiological and evolutionary mechanisms of human influenza, our model nevertheless incorporates a substantial number of parameters. We focus on the following groupings:

— the seasonal peak value of *R*_0_, here termed *R*_p_, and its relative amplitude *δ* (*R*_p_(1 – *δ*) being the seasonal minimum value of *R*_0_). Strictly, these parameters control overall transmissibility and the magnitude of seasonal forcing of transmission;
— the timescale for the generation of antigenically new strains, represented by mean duration of immunity to the current influenza strain, *D*, and the cross-immunity between strains, *ϕ* (§2);
— the degree of assortativity in the contact patterns between children and adults, *θ*;
— external force of infection, *ε*, representing effect of contact between members of the modelled population and infected individuals outside the modelled population.

The values of other parameters are listed in table 1 and discussed in the electronic supplementary material. In discussing the behaviour of the model, periodicity refers to the periodicity of the overall case rate with time, rather than for an individual strain.

**Table 1.** Baseline model parameter values. Values shown are those used if not otherwise specified.

parameter	symbol	value
birth–death rate	*μ*	1/70 yr^−1^
child age class width	*L*_c_	14 year
adult age class width	*L*_a_	56 year
mean generation time	1/*α*	2.7 days
generation time shape parameter	*M*	4 days
relative amplitude	*δ*	0.2 days
relative infectiousness	*ψ*	1.5 days
relative susceptibility	*ρ*	2 days
mixing parameter	*θ*	0.3 days
immunity duration	*D*	5 years
total population	*N*	6 × 10^7^
cross-immunity	*φ*	0.25
external force of infection (FOI)	*ε*	2 × 10^−6^ yr^−1^

Simulations were run using a population of 60 million, approximating the population of the UK. Owing to the large population, a deterministic model was used. Tests using the corresponding stochastic models showed no qualitative variation from the deterministic dynamics and the presence of a continuous low level external force of infection precluded the possibility of extinction.

## 3. Results

**Figure 3.**
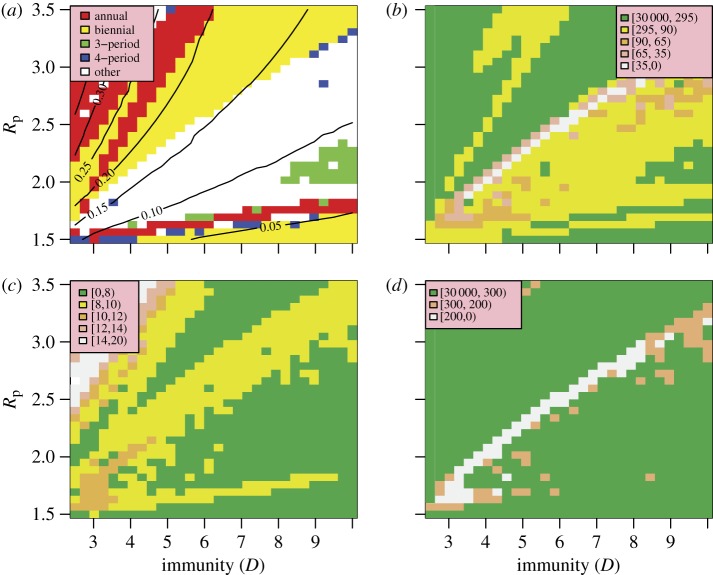
Model behaviour as a function of peak *R*_p_ and duration of immunity, *D*. (*a*) Periodicity of model dynamics (colours) and annual attack rate (contours). Periodicities of 5 years and greater are grouped as ‘other’; (*b*) quality of fit as information distance (ID) for annual attack rate (AAR); (*c*) annual duration of epidemic (ADE) in weeks; (*d*) quality of fit (ID) to annual attack rate and epidemic duration combined. Other parameters as given in table 1.

**Figure 4.**
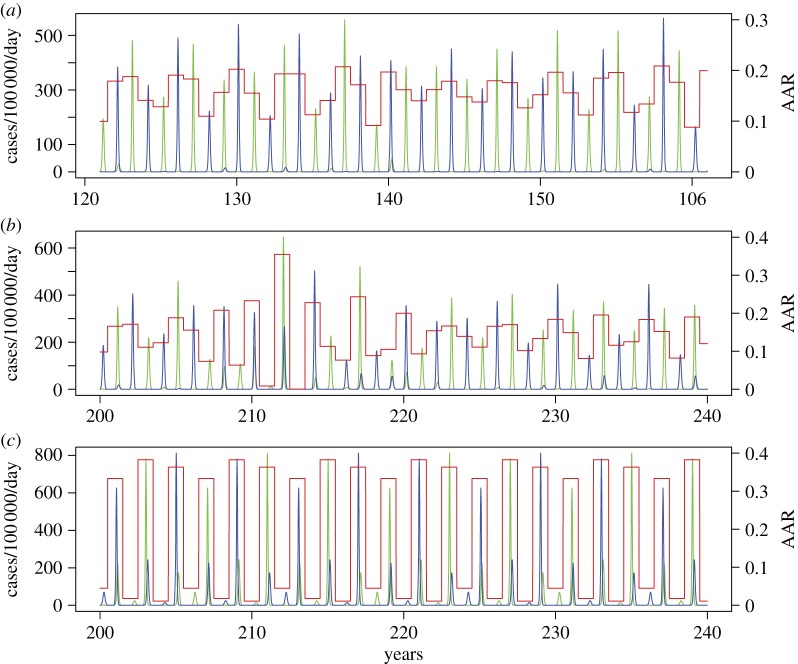
Model output time series of case incidence (the two subtypes being represented by blue and green) and annual attack rates (red) for the parameter combination used in figure 2. (*a*) *R*_p_ = 2.0, *D* = 4 yr^−1^: KL distance = 115. (*b*) *R*_p_ = 1.85, *D* = 3.7 yr^−1^: KL distance = 227. (*c*) *R*_p_ = 2.0, *D* = 3.36 yr^−1^: KL distance = 1116.

Figure 3 illustrates the behaviour of the model and aspects of the information distance between model and data as a function of *R*_p_ and *D*. The region of best fits is located in a narrow diagonal band spanning 1.6 < *R*_p_ < 2.5 and 3 < *D* < 8 (figure 3*d*). Along this band, increasing reproduction number is compensated for by a longer period of effective immunity that decreases the susceptible proportion of the population, giving a constant mean attack rate. The acceptable region is bounded in part by the duration of the model epidemics. The lowest and highest values of *R*_p_ generate epidemics that are too broad and too narrow, compared with the target distribution. The fit of the model is strongly constrained by the dynamics of the model, which exhibits a wide range of periodic behaviour across quite small changes in parameter values (figure 3*a*). While AAR changes smoothly with the parameters, abrupt changes in periodicity lead to qualitative changes in the distributions of AAR and epidemic duration and hence the KL distance. Best-fit behaviour is associated with long-period behaviour of the model (4+ years). Here, the model generates a range of AARs clustered around the mean and matching the target distribution. The qualitatively different forms of behaviour are well characterized by the total KL distance measure (figure 4). KL distances less than 200 correspond to realistic behaviour with appropriate mean attack rate distributions (figure 4*a*). KL distance between 200 and 300 match either with realistic behaviour interspersed with large-scale epidemic episodes or with realistic behaviour but with a mean attack rate displaced from the target value (figure 4*b*). Larger values represent dynamics and mean attack rates greatly different from the observed time series (figure 4*c*).

**Figure 5.**
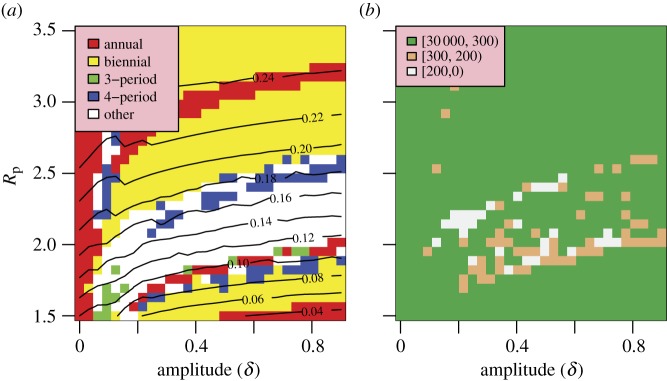
Model fit as a function of *R*_p_ and *δ* (*D* = 4.5 yr). (*a*) Periodicity of the model. (*b*) Total information distance.

Figure 5 illustrates a strong sensitivity to the amplitude of variation of the contact parameter, *δ*, with the best-fit lying in the range 0.15–0.3. Although the mean AAR is not strongly dependent on *δ* (figure 5*a*), the bifurcation behaviour of the model means realistic solutions (resembling figure 4*a*,*b*) can be found for higher amplitudes of seasonal variation but not in a contiguous region (figure 5*b*). Solutions with smaller seasonal variations are rejected on the epidemic duration component of the information distance. Low amplitudes generate broad epidemics which do not match the target distribution.

**Figure 6.**
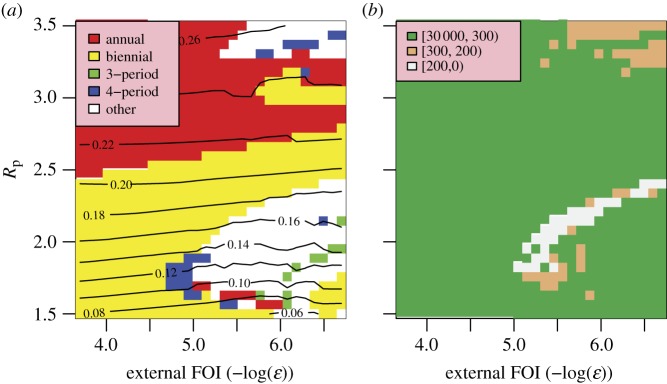
Model fit as a function of *R*_p_ and external forcing. (*a*) Periodicity of the model. (*b*) Total information distance (note forcing increases to the left).

The behaviour of our model is quite sensitive to the assumed external force of infection, *ε*. The level of external forcing assumed for most of this work (§2) is negligible compared with the average force of infection generated by the indigenous population. However, long-period and chaotic solutions for seasonally forced SIR models generate very low infection prevalence during epidemic troughs, even low levels of importation of infectives strongly encourages annual and biennial behaviours and removes highly chaotic solutions from the optimal region. The effect of importation rate can be seen in figure 6. For external forces of infection above about 10^−5^ yr^–1^, only annual and biennial solutions are found. Optimal behaviour is found for an external force of infection of approximately 6 × 10^−7^ yr^–1^.

**Figure 7.**
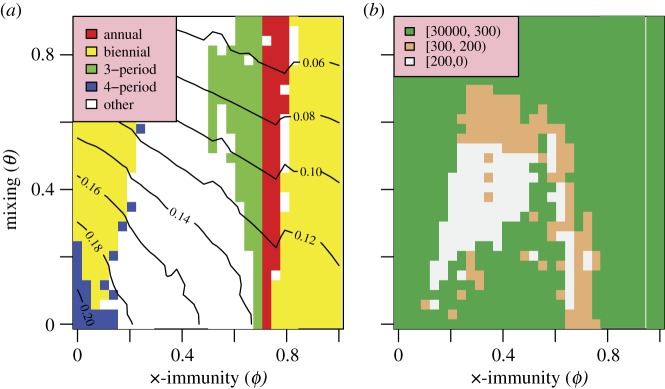
Model fit as a function of population heterogeneity and cross-immunity. (*a*) Periodicity of the model. (*b*) Total information distance.

Figure 7 explores the sensitivity of the model to the degree of heterogeneity and cross-immunity in the population. The choice of *R*_p_ and *D* lies in the well-fitting band in figure 3*d*. It is clear that a wide range of values for these parameters allow the model to fit the behaviour of the time series quite well. The model's qualitative behaviour (in terms of its periodicity) is more stable with respect to these mechanisms and variation within the closest fit region mainly affects the mean attack rate. There is a broadly inverse relationship between the well-fitting values of the parameters *θ* and *ϕ*. Increasing the assortativity of mixing concentrates infections more strongly in age groups, decreasing the available susceptibles and hence the attack rate. Increasing cross-immunity has an equivalent effect by increasing the effective loss of susceptibles caused by any single infection event and hence reducing the attack rate. As can be seen from figure 7*a*, values of the cross-immunity parameter *ϕ* outside the range 0.3–0.6 drive the model into unfavourable periodicities, giving very poor fits. This suggests that a model with two strains interacting via cross-immunity is necessary to reproduce the dynamics seen in influenza time series and that it is insufficient to have two independent strains (*ϕ* = 0) or two antigenically identical strains (i.e. *ϕ* = 1−equivalent to a single strain model). Similarly, extreme values of *θ* also lead to poorly fitting model behaviour, suggesting that a uniformly mixing population (*θ* = 0) would also not generate matching behaviour.

## 4. Discussion

In this work, we have identified a minimal set of mechanisms necessary to match the long-term temporal behaviour seen in ILI time series. We find that an age-structured population and multiple strains with cross-immunity are necessary to recreate the distributions of AAR and epidemic duration seen in time-series data. Nonlinear models of this type with temporal forcing are well known for having complex bifurcation structures affecting their periodic behaviour. On the timescale of a single disease season or single epidemic, these would have little effect on parameter estimation. Over many seasons, however, the periodicity of the underlying model is crucially important. We would argue that capturing the long-term trends in behaviour is at least as important as the detail of individual seasonal outbreaks and our fitting approach focuses on these aspects.

The complex bifurcation structure of the model means that the information distance is not necessarily a smooth function of the parameters. This makes finding a unique set of parameters giving an overall minimum distance impossible. Although mean attack rate and duration generally vary smoothly, the period of the model solution changes discontinuously (inevitably, as it only takes values that are multiples of the annual forcing period). Solutions with longer periods generate a wider range of AAR and epidemic durations over an extended period of time and are therefore capable of fitting the target data distributions better. As a result, the best fits are strongly associated with longer periods in model solutions and the quality of fit of the model can change abruptly over small changes in parameter values.

Peak *R*_0_, the amplitude of variation of *R*_0_ and the duration of immunity are all strongly constrained. Figure 3 illustrates that increasing *R*_0_ is offset by a longer duration of immunity reducing the susceptible population. The narrowness of the well-fitting parameter region is a result of the sudden changes of behaviour generated by changes in these parameters. Figure 5 also illustrates this feature. The closest fitting region is found for *δ* between 0.15 and 0.3, but patches of well-fitting solutions are scattered a range of values of *R*_p_ and *δ* owing to the sensitivity to the system to temporal forcing. We note that a change in the mode of forcing from sinusoidal to school-term leads to generally broader ranges of acceptable parameter values (see the electronic supplementary material), perhaps indicating that the presence of this mechanism is a strong contributor to the variable annual behaviour observed in the ILI dataset.

Because of the difficulties in knowing the ‘true’ incidence rate, the mean AAR is not precisely known and a range of 10–20% is often quoted. To allow for this uncertainty, we investigated allowing the information distance calculation to be based on the best-fit mean AAR from the range 10–20%, rather than precisely 15 per cent per year. Resulting best-fit parameter regions for *R*_0_ against *D* and *θ* against *ϕ* were not significantly changed, owing almost certainly to the dominance of qualitative model behaviour as described above.

Incidence periodicity of the model is much less sensitive to cross-immunity and age structure, resulting in a wide region of close fitting behaviour for values of *θ* between about 0.2 and 0.6 and *ϕ* between 0.3 and 0.5. As discussed in §3, this strongly suggests that an age-structured population and, in particular, a pathogen population with more than one strain and cross-immunity, are necessary to reproduce the patterns of behaviour found in the ILI time series. Models based on single strains and well-mixed populations generate annual and biennial behaviour for the same parameter values. The necessity of multiple strains has been noted in other work modelling seasonal influenza [14], although in that work the two strains did not interact.

Within the best-fitting parameter regimes, incidence periodicity for each modelled strain is basically biennial with strains dominating alternate years. While real strain dynamics are clearly more complex than this (figure 1), observed patterns do show a tendency for a particular strain not to dominate in successive years. Within the model, cross-immunity generates a negative correlation between strains, causing them to alternate in successive years. For strong cross-immunity, both strains become antigenically similar and goodness of fit falls off rapidly.

It is instructive to compare our results with those of previous papers fitting simple models to influenza incidence data. Work by Xia *et al*. [15] used a simple single strain SIRS, but with a more detailed description of temporal variation in contact rate and loss of immunity post-recovery. In addition, the infection rate was described by the phenomenological term *β* *I*^*α*^ *g*(*S*). Exponents of this type are well known to facilitate fitting [5], but are hard to interpret. We note that both the form of this term and the non-exponentially distributed duration of immunity in that study affect epidemic attack rates as a function of transmissibility and the periodicity of epidemics, and hence may play an equivalent role to age structure and the incorporation of two subtypes in our model in allowing a good fit to the data.

Shaman *et al*. [14] use a similar SIRS model to investigate the possibility that seasonal changes in absolute humidity can generate recorded patterns in pneumonia and influenza mortality data. The model was stochastic, has no age structure, and effectively uses only one strain. Best-fit parameter values are similar to those found in this work, although the relative amplitude of variation in *R*_0_ is in the range 0.4–0.5, significantly higher than our findings. Best-fit parameter sets showed considerable lack of correlation with each other, which the authors attribute to the stochastic nature of their model. Our work suggests that this scatter may be the result of the complex bifurcational structure of such models. As already discussed, our results indicate that a two strain model without cross-immunity or age structure is unlikely to fit patterns of seasonal influenza from a temperate region. However, there are several significant differences between the two systems. Shaman *et al*. employ a significantly higher background force of infection than ours (approx. 7.3 × 10^−4^ yr^–1^), which would place our model in a strongly annual or biennial regime. Hence that model may reproduce the mean attack rate well, but not its variability.

Our assessment of goodness of fit is currently focused primarily on distribution of AARs and duration of epidemics, although we also take account of the sequence of strains and the age-distribution of cases (see the electronic supplementary material). Future work will test our conclusions against a full description of the incidence data as well as against different choices of key features in the data, such as time of epidemic onset.
